# circ_CHFR regulates ox-LDL-mediated cell proliferation, apoptosis, and EndoMT by miR-15a-5p/EGFR axis in human brain microvessel endothelial cells

**DOI:** 10.1515/biol-2021-0082

**Published:** 2021-09-29

**Authors:** Shanwu Wu, Sheng Yang, Hongyan Qu

**Affiliations:** Department of Neurosurgery, Sinopharm Dongfeng General Hospital, No. 16 Daling Road, Zhangwan District, Shiyan City, 442000, Hubei, China

**Keywords:** ox-LDL, circ_CHFR, miR-15a-5p, EGFR, HBMECs

## Abstract

Oxidized low-density lipoprotein (ox-LDL) is a significant risk factor for various brain vascular diseases. Circular RNA (circRNA) is involved in the pathogenesis of brain vascular diseases. This study revealed the roles of circ_CHFR in ox-LDL-mediated cell proliferation, apoptosis, and endothelial-to-mesenchymal transition (EndoMT). Our results showed that circ_CHFR and EGFR expressions were dramatically upregulated, while miR-15a-5p expression was downregulated in ox-LDL-induced human brain microvessel endothelial cells (HBMECs) relative to control groups. circ_CHFR knockdown hindered the effects of ox-LDL exposure on cell proliferation, cell cycle, apoptosis, and EndoMT in HBMECs, whereas these impacts were abolished by miR-15a-5p inhibitor. In addition, circ_CHFR functioned as a sponge of miR-15a-5p and miR-15a-5p bound to EGFR. Thus, we concluded that circ_CHFR silencing hindered ox-LDL-mediated cell proliferation, apoptosis, and EndoMT by downregulating EGFR expression through sponging miR-15a-5p in HBMECs. Our findings provide a new mechanism for studying circRNA-directed therapy in ox-LDL-induced human brain vascular diseases.

## 1 Introduction

Endothelial cells are vital cells in regulating the function and the structure of vessels [1]. Therefore, endothelial cell dysfunction is regarded as a universal physiological phenomenon of vascular diseases [2]. Brain endothelial cells play a key part in modulating blood–brain barrier (BBB) function and cerebrovascular homeostasis, and their dysregulation is considered a primary cause of cerebrovascular diseases [3,4,5]. Oxidized low-density lipoprotein (ox-LDL) is a crucial inducement of cerebrovascular diseases based on its ability in increasing oxidative stress, upregulating the number of inflammatory factors and abnormally modulating cell proliferation and migration [6,7,8]. In inflammatory reactions, mediators and cytokines, as well as inflammatory cells, play an important role [9]. However, the pathogenesis of cerebrovascular diseases induced by ox-LDL has not been completely demonstrated.

Circular RNA (circRNA) is a stable noncoding RNA with its closed-loop structure [10,11]. Previous studies have illustrated that circRNA is widely expressed in many organs, especially in the brain [12]. For example, Peng et al. explained that circRNA HECT domain E3 ubiquitin protein ligase 1 (circ_HECTD1) was highly expressed in acute ischemic stroke (AIS) and could be employed as a biomarker in distinguishing AIS with other diseases [13]. Shen et al. also illustrated that circ-0044073 was highly expressed and contributed to cell proliferation and invasion in atherosclerosis [14]. The aforementioned data suggest that circRNA may be enrolled in the pathogenic mechanism of brain-related diseases. In addition, circRNA was disclosed to regulate ox-LDL-induced deleterious effects in endothelial cells. For instance, Li et al. proved that circ_0003575 modulated ox-LDL-induced cell proliferation and angiogenesis in endothelial cells [15]. Qin et al. presented that circ_0003645 knockdown hindered cell apoptosis and inflammation induced by ox-LDL in endothelial cells [16]. Nevertheless, there are no data on regulating ox-LDL-induced human brain microvessel endothelial cells (HBMECs) injury by circ_CHFR.

microRNA (miRNA) is a small noncoding RNA and regulates transcriptional and posttranscriptional processes [17]. miRNAs have been indicated to modulate many biological processes in cells, such as proliferation, apoptosis, and differentiation [18]. Existed researches also explained that miRNAs were involved in endothelial cell development. For example, miR-17 was reported to accelerate cell proliferation and suppress cell apoptosis in endothelial cells [19]. Li et al. elucidated that miR-210 accelerated endothelial cell apoptosis in atherosclerosis [20]. However, the effects of miR-15a-5p on HBMEC development are unknown. Epidermal growth factor receptor (EGFR), one of the receptor tyrosine kinases, has been unveiled to participate in physiological and pathological processes of vessels [21]. In addition, Fu et al. also explained that EGFR facilitated the invasion of BMEC via recruiting actinin-4 [22]. These pieces of evidence demonstrate that EGFR may play a vital part in HBMEC development.

Herein, the expression profiles of circ_CHFR, miR-15a-5p, and EGFR were determined in ox-LDL-induced HBMECs. In addition, the effects of ox-LDL treatment on the proliferation, apoptosis, and endothelial-to-mesenchymal transition (EndoMT) were disclosed. Furthermore, rescue experiments were employed to illustrate that circ_CHFR knockdown regulated ox-LDL-mediated cell proliferation, apoptosis, and EndoMT by downregulating EGFR expression through binding to miR-15a-5p.

## 2 Materials and methods

### 2.1 Cell acquirement and culture

HBMECs were purchased from Otwo Biotech (Shenzhen, China). HBMECs were cultured in Dulbecco’s modified Eagle’s medium (DMEM; HyClone, Logan, UT, USA) with 10% fetal bovine serum (FBS; HyClone) and antibiotics (100 μg/mL penicillin, 100 μg/mL streptomycin; Gibco, Carlsbad, CA, USA) at 37°C in an incubator with 5% CO_2_.

### 2.2 Plasmid construction and cell transfection

The small-interfering RNA against circ_CHFR (si-circ_CHFR), miR-15a-5p mimic (miR-15a-5p), miR-15a-5p inhibitor (anti-miR-15a-5p), the overexpression plasmid of EGFR (EGFR), and control groups (si-NC, miR-NC, anti-miR-NC, and pcDNA) were synthesized by GENEWIZ Co., Ltd. (Suzhou, China). Cell transfection was performed using Lipofectamine 2000 (Thermo Fisher, Waltham, MA, USA) as previously described [23]. The synthesized sequences were si-circ_CHFR 5′-CTCAGCAGTCCAGCCATACGT-3′, miR-15a-5p mimic 5′-UAGCAGCACAUAAUGGUUUGUG-3′, miR-15a-5p inhibitor 5′-CACAAACCAUUAUGUGCUGCUA-3′, si-NC 5′-CCAACCAGTTAACTCGAAT-3′, miR-NC 5′-UUUGUACUACACAAAAGUACUG-3′, and anti-miR-NC 5′-CAGUACUUUUGUGUAGUACAAA-3′.

### 2.3 Quantitative reverse transcription polymerase chain reaction (qRT-PCR)

Ox-LDL-induced HBMECs were collected and lysed using TransZol (TransGen, Beijing, China). RNA was reversely transcribed into cDNA with a High-Capacity cDNA RT Kit (Thermo Fisher) or MiX-x™ synthesis Kit (TaKaRa, Dalian, China). To determine the expression of circ_CHFR, checkpoint with forkhead and ring finger domains (CHFR), miR-15a-5p, and EGFR, SuperReal PreMix (Tiangen, Beijing, China) was employed. Data were analyzed with the 2^−∆∆Ct^ method with U6 and glyceraldehyde 3-phosphate dehydrogenase (GAPDH) as references. The sequences of forward and reverse primers were circ_CHFR 5′-CCCTCTGCAAGGAAGCCACG-3′ and 5′-TGCGCCGCCTGCCTTCTGTA-3′; CHFR 5′-CTCGTGTTGGGCTCGTGTC-3′ and 5′-GAGCAGGTTTCACAGGAGTCA-3′; miR-15a-5p 5′-CTCACGTAGCAGCACATAA-3′ and 5′-ACCTCAAGAACAGTATTTCCAGG-3′; EGFR 5′-GACGACAGGCCACCTCG-3′ and 5′-ATCGCTGCTCCCCGAAGA-3′; U6 5′-CTCGCTTCGGCAGCACATATACT-3′ and 5′-ACGCTTCACGAATTTGCGTGTC-3′; GAPDH 5′-AACGGATTTGGTCGTATTGGG-3′ and 5′-CGCTCCTGGAAGATGGTGAT-3′.

### 2.4 RNase R and actinomycin D treatment assays

The two types of assays were performed following the previously shown method [24]. In short, HBMECs were collected and lysed using TransZol (TransGen), and RNA was isolated in the same manner as shown earlier. Extracted RNA was incubated with RNase R (Epicentre, Madison, WI, USA) at 37°C for 30 min. RNeasy MinElute Cleaning Kit (Qiagen, Valencia, CA, USA) was employed to purify RNA. In addition, HBMECs were incubated with actinomycin D (Millipore, Bradford, MA, USA) for 0, 4, 8, 16, and 24 h to block RNA synthesis. The levels of circ_CHFR and CHFR were determined by quantitative reverse transcription polymerase chain reaction (qRT-PCR). CHFR was used as a control.

### 2.5 Cell cycle and apoptosis analysis

Cell cycle and cell apoptosis were investigated with Cell Cycle and Apoptosis Analysis Kit (Yeasen Biotech, Shanghai, China) according to manufacturer’s instructions. In short, cells were treated with 50 μg/mL ox-LDL (Solarbio, Beijing, China) and transfected. Forty-eight hours later, the medium was discarded, and cells were collected. Then, cells were washed using cold phosphate-buffered solution (PBS; Thermo Fisher). Cells were fixed with 70% ethanol (Millipore) overnight. Following that, cells were incubated with Annexin V-fluorescein isothiocyanate (Annexin V-FITC; Yeasen Biotech), propidium iodide (PI; Yeasen Biotech), or RNase A (Yeasen Biotech) at 37°C for 30 min. Finally, cell cycle process and apoptosis were analyzed using flow cytometry (BD Biosciences, San Diego, CA, USA).

### 2.6 3-(4,5)-Dimethylthiahiazo(-z-y1)-3,5-di-phenytetrazoliumromide assay

3-(4,5)-Dimethylthiahiazo(-z-y1)-3,5-di-phenytetrazoliumromide (MTT) assay was carried out with the MTT kit (Beyotime, Shanghai, China) according to the instructions of the manufacture. In brief, HBMECs were seeded in a 96-well plate for 24 h. The medium was removed, and fresh DMEM mixed with 10% FBS (HyClone, Logan, UT, USA) was added. 50 μg/mL ox-LDL (Solarbio) was exposed into the wells, and cell transfection was performed. Cells were continued to be cultured for 1, 2, and 3 days. Then, MTT solution (Beyotime) was added, and cells were cultivated for another 4 h. The medium was discarded, and dimethyl sulfoxide (DMSO; Sigma, St. Louis, MO, USA) was exposed to the plate to dissolve formazan crystal. Cell proliferation was detected by measuring absorbance at 570 nm using a microplate reader (Thermo Fisher).

### 2.7 Western blot analysis

Cells were harvested after various treatments. Western blot analysis was conducted based on the published procedures [25]. Briefly, lysis buffer (Beyotime) was used to lyse cells. Lysates were mixed with loading buffer (Thermo Fisher), which was then boiled in boiling water for 8 min. The protein sample was loaded on 12% bis-tris-acrylamide gel (Thermo Fisher). Then, the protein bands were electrotransferred onto polyvinylidene fluoride (Millipore) and then immersed in 5% nonfat milk (Solarbio). Following that, the membranes were incubated with primary antibodies at 4°C overnight and secondary antibodies (peroxidase-conjugated IgG; 1:1,000; Abcam, Cambridge, UK) at 37°C for 2 h. Protein bands were visualized with eyoECL Plus Kit (Beyotime). GAPDH was employed as a control. Primary antibodies were anti-Ki67 antigen (anti-Ki67; 1:250; Abcam), anti-B-cell lymphoma-2 (anti-Bcl-2; 1:1,000; Abcam), anti-BCL2-associated x protein (anti-Bax; 1:1,000; Abcam), anti-Cleaved poly (ADP-ribose) polymerase (PARP) (anti-Cleaved PARP; 1:1,000; CST, Boston, MA, USA), anti-collagen typeI2 (anti-COL1A2; 1:1,000; Abcam), anti-actin alpha 2 (anti-ACTA2; 1:500; Abcam), and anti-GAPDH (1:1,000; CST).

### 2.8 Dual-luciferase reporter assay

The binding sites between miR-15a-5p and circ_CHFR or EGFR were predicted by the starbase3.0 online database. The wild-type (WT) plasmids of circ_CHFR (circ_CHFR-WT) and EGFR (EGFR-WT) were built by inserting the sequences of circ_CHFR and the 3′-untranslated regions (3′UTR) of EGFR into the pmirGLO vector (Promega, Madison, WI, USA). The mutant (MUT) plasmids of circ_CHFR (circ_CHFR-MUT) and EGFR (EGFR-MUT) were constructed by subcloning the mutant sequences of circ_CHFR and EGFR 3′UTR into pmirGLO vector (Promega). Plasmids were transfected into cells with miR-15a-5p mimic or miR-NC. Luciferase activities were detected using Dual-Lucy Assay Kit (Solarbio) as per the guidebook. *Ranilla* luciferase activity acted as a control.

### 2.9 Data analysis

Data were assessed by SPSS 21.0 software (IBM, Somers, NY, USA) based on three replicates. Data were presented as means ± standard deviations (SDs). Significant differences were compared via two-tailed Student’s *t*-tests or one-way analysis of variance (ANOVA). *P* < 0.05 was considered statistically significant.

## 3 Results

### 3.1 circ_CHFR expression was dramatically upregulated in ox-LDL-induced HBMECs

**Figure 1:**
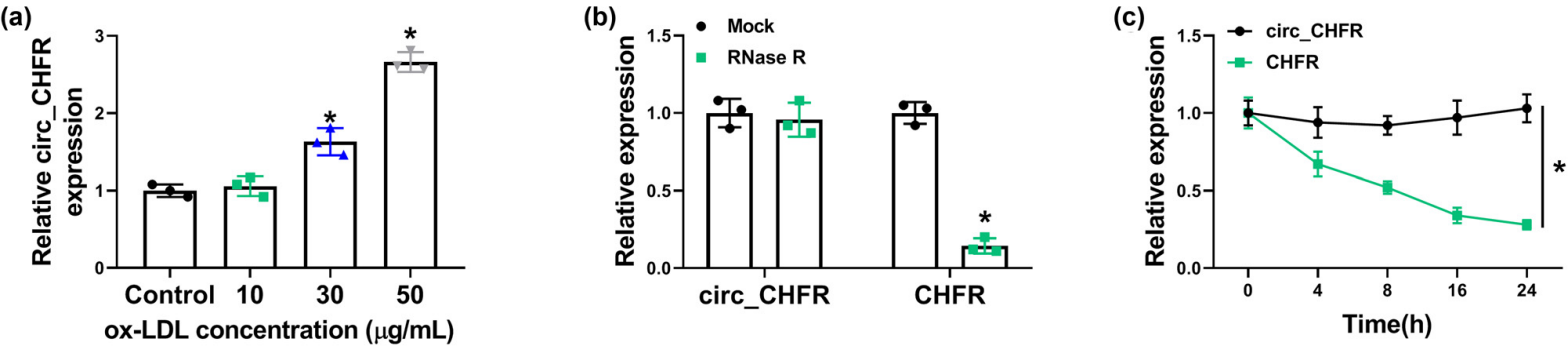
circ_CHFR was highly expressed in HBMECs treated with ox-LDL. (a) circ_CHFR expression was detected by qRT-PCR in HBMECs treated with ox-LDL (10, 30 and 50 μg/mL). (b and c) RNase R and actinomycin D treatment assays were employed to demonstrate circ_CHFR was a circular RNA. *P < 0.05.

The effect of ox-LDL treatment (10, 30, and 50 μg/mL) on circ_CHFR expression was first detected. Results showed that circ_CHFR expression was upregulated by ox-LDL (30 and 50 μg/mL; Figure 1a), especially by 50 μg/mL ox-LDL. Based on the aforementioned data, cells were treated with 50 μg/mL ox-LDL in a further study. Subsequently, results presented that the content of circ_CHFR was not changed after RNase R treatment, whereas the expression of its linear form (CHFR) was significantly downregulated (Figure 1b). Meanwhile, data also displayed that circ_CHFR expression level was not obviously changed after actinomycin D exposure, and CHFR expression was significantly reduced (Figure 1c). Thus, these results demonstrated that circ_CHFR was more stable than linear RNA, and ox-LDL treatment upregulated circ_CHFR expression in HBMECs.

### 3.2 circ_CHFR knockdown hindered the effects of ox-LDL treatment on cell proliferation, apoptosis, and EndoMT in HBMECs

**Figure 2:**
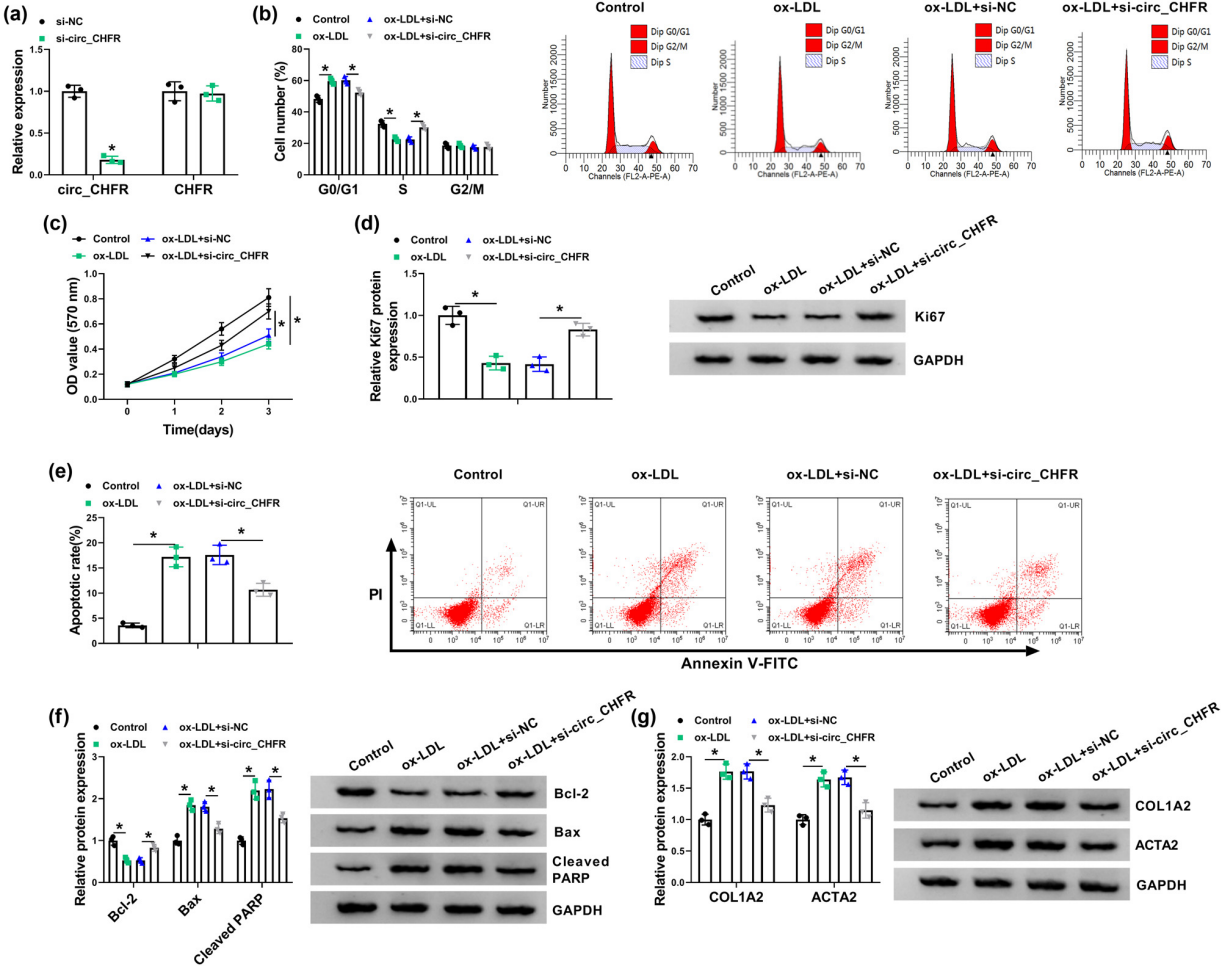
circ_CHFR silencing abolished the impacts of ox-LDL treatment on the biological behaviors of HBMECs. (a) The knockdown efficiency of si-circ_CHFR was determined by qRT-PCR. (b) The effects between ox-LDL treatment and circ_CHFR silencing on cell cycle process were disclosed by cell cycle assay in HBMECs. (c) The effects between ox-LDL treatment and circ_CHFR knockdown on the proliferation of HBMECs were investigated by MTT assay. (d, f, and g) Western blot was employed to explain the impacts between ox-LDL treatment and circ_CHFR repression on the protein expression of Ki67, Bcl-2, Bax, cleaved PARP, COL1A2, and ACTA2 in HBMECs. (e) Cell apoptosis assay was conducted to unveil the influences between ox-LDL exposure and circ_CHFR silencing on the apoptosis of HBMECs. *P < 0.05.

Whether circ_CHFR could mediate the effects of ox-LDL treatment on the biological behaviors of HBMECs was further studied. Results first illustrated that circ_CHFR expression was dramatically downregulated in HBMECs transfected with si-circ_CHFR, whereas there was no obvious change in CHFR expression (Figure 2a) after circ_CHFR silencing, meaning that the interfering plasmid of circ_CHFR was successfully built. Subsequently, data disclosed that cell cycle arrested at G0/G1 phase after ox-LDL treatment in HBMECs, whereas this effect was attenuated by circ_CHFR silencing (Figure 2b). The proliferation of HBMECs was also inhibited by ox-LDL treatment, but circ_CHFR silencing reversed this impact (Figure 2c). Western blot showed that the expression of proliferation-related protein Ki67 was inhibited after ox-LDL treatment in HBMECs; however, this effect was restored by circ_CHFR silencing (Figure 2d). In addition, results displayed that ox-LDL treatment-induced cell apoptosis, which was restrained by circ_CHFR depletion (Figure 2e). Western blot results also displayed that the protein expression of Bcl-2 was dramatically downregulated, and the protein expression of Bax, cleaved PARP, COL1A2, and ACTA2 was obviously upregulated in HBMECs treated with ox-LDL; however, these influences were abolished by circ_CHFR knockdown (Figure 2f and g). The aforementioned data demonstrated that circ_CHFR silencing could protect against ox-LDL-induced cell injury.

### 3.3 circ_CHFR was a sponge of miR-15a-5p in HBMECs

**Figure 3:**
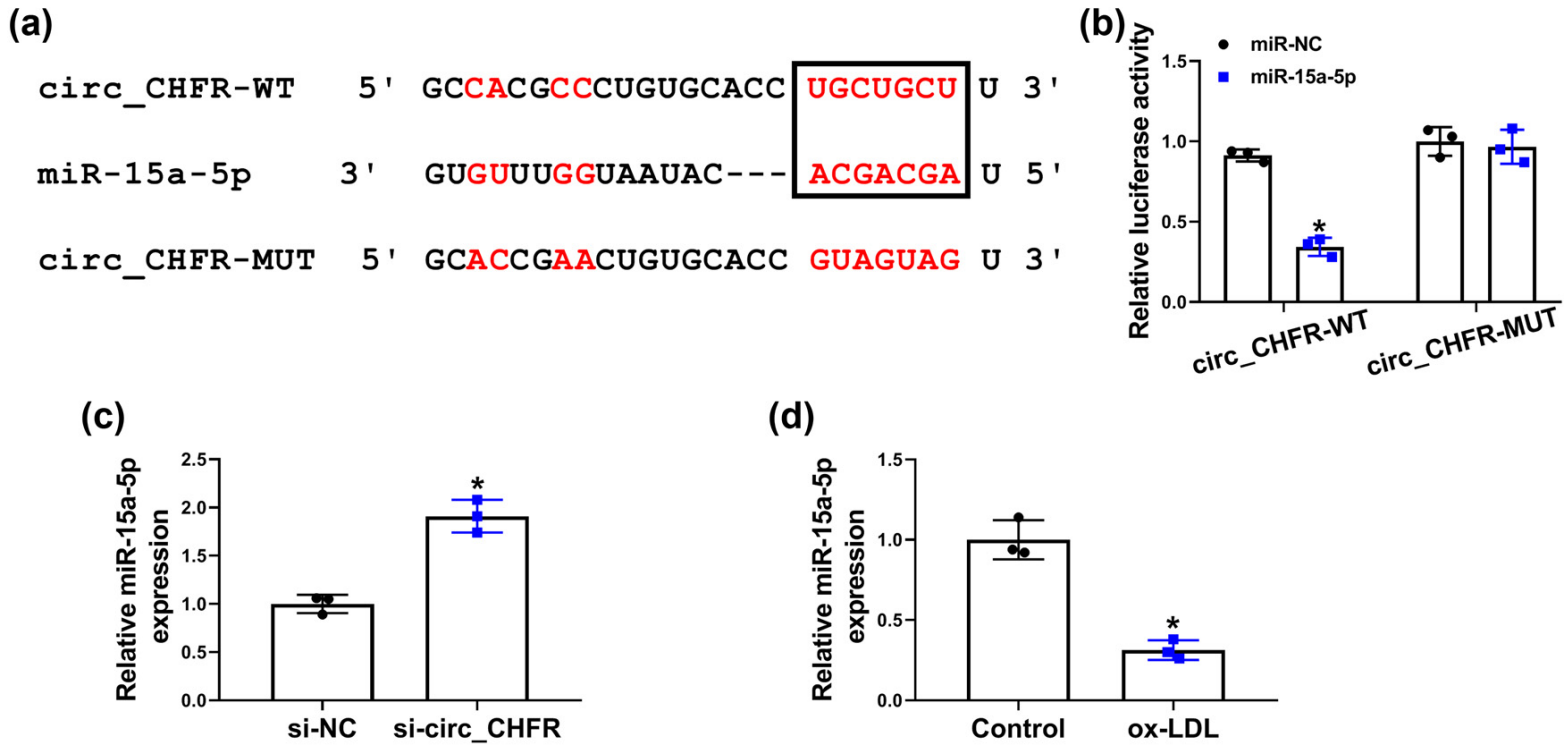
circ_CHFR functioned as a sponge of miR-15a-5p in HBMECs. (a) The binding sites between miR-15a-5p and circ_CHFR were predicted by starbase3.0 online database. (b) Luciferase activity was determined by dual-luciferase reporter assay in HBMECs. (c and d) QRT-PCR was employed to detect the impacts of circ_CHFR silencing and ox-LDL treatment on miR-15a-5p expression in HBMECs. *P < 0.05.

The underneath mechanism of circ_CHFR in regulating ox-LDL-mediated biological behaviors of HBMECs was revealed in this part. Starbase3.0 online database showed that miR-15a-5p contained the binding sites of circ_CHFR (Figure 3a). Dual-luciferase reporter assay demonstrated that the luciferase activity of circ_CHFR-WT and miR-15a-5p group was dramatically repressed, whereas there was no obvious change in the luciferase activity of circ_CHFR-MUT and miR-15a-5p group (Figure 3b). Subsequently, results showed that miR-15a-5p expression was dramatically upregulated by circ_CHFR knockdown and downregulated after ox-LDL treatment (Figure 3c and d). These data explained that circ_CHFR was associated with miR-15a-5p.

### 3.4 circ_CHFR regulated ox-LDL-medicated cell proliferation, apoptosis, and EndoMT by sponging miR-15a-5p in HBMECs

**Figure 4:**
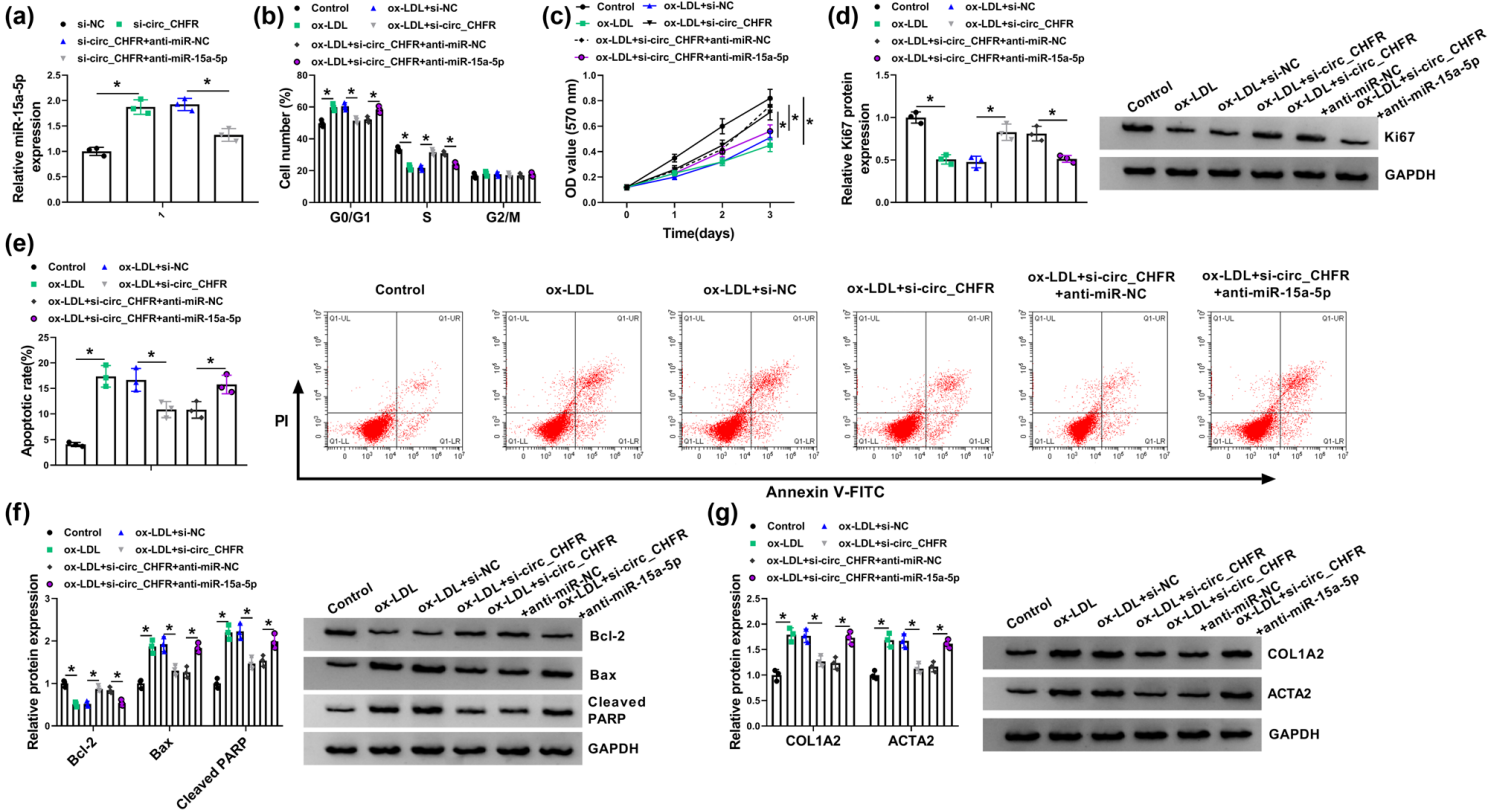
circ_CHFR regulated ox-LDL-medicated cell proliferation, apoptosis, and EndoMT by interacting with miR-15a-5p in HBMECs. (a) The effects between circ_CHFR silencing and miR-15a-5p inhibitor on miR-15a-5p expression were demonstrated by qRT-PCR. (b and c) Cell cycle and MTT assays were employed to determine the influences between circ_CHFR silencing and miR-15a-5p inhibitor on cell proliferation under ox-LDL treatment in HBMECs. (d, f, and g) Western blot was employed to explain the impacts between circ_CHFR repression and miR-15a-5p inhibitor on the protein expression of Ki67, Bcl-2, Bax, cleaved PARP, COL1A2, and ACTA2 after ox-LDL treatment in HBMECs. (e) Cell apoptosis assay was carried out to explain the impacts between circ_CHFR silencing and miR-15a-5p inhibitor on the apoptosis of HBMECs after ox-LDL treatment. *P < 0.05.

To determine whether circ_CHFR regulated ox-LDL-medicated cell proliferation, apoptosis, and EndoMT by associating with miR-15a-5p, loss-of-function experiments were employed. Results first showed that miR-15a-5p expression was significantly upregulated by circ_CHFR knockdown, whereas this effect was attenuated after transfection of miR-15a-5p inhibitor (Figure 4a). Subsequently, it was found that circ_CHFR silencing restored the promoting effect of ox-LDL treatment on cell cycle arrest, whereas this effect was hindered by miR-15a-5p inhibitor (Figure 4b). MTT assay also demonstrated that miR-15a-5p inhibitor attenuated the promoting effect of circ_CHFR knockdown on cell proliferation under ox-LDL treatment (Figure 4c). Also, western blot showed that Ki67 protein expression was upregulated by circ_CHFR knockdown after ox-LDL treatment, which was restrained after miR-15a-5p inhibitor transfection (Figure 4d). In addition, the apoptosis of HBMECs was repressed by circ_CHFR knockdown under ox-LDL treatment; however, miR-15a-5p inhibitor relieved this influence (Figure 4e). The protein expression of Bcl-2 was upregulated, and the protein expression of Bax, cleaved PARP, COL1A2, and ACTA2 was downregulated by circ_CHFR silencing after ox-LDL treatment, but miR-15a-5p inhibitor abolished these effects (Figure 4f and g). All these pieces of evidence demonstrated that circ_CHFR regulated ox-LDL-mediated cell proliferation, apoptosis, and EndoMT by binding to miR-15a-5p in HBMECs.

### 3.5 miR-15a-5p was associated with EGFR in HBMECs

**Figure 5:**
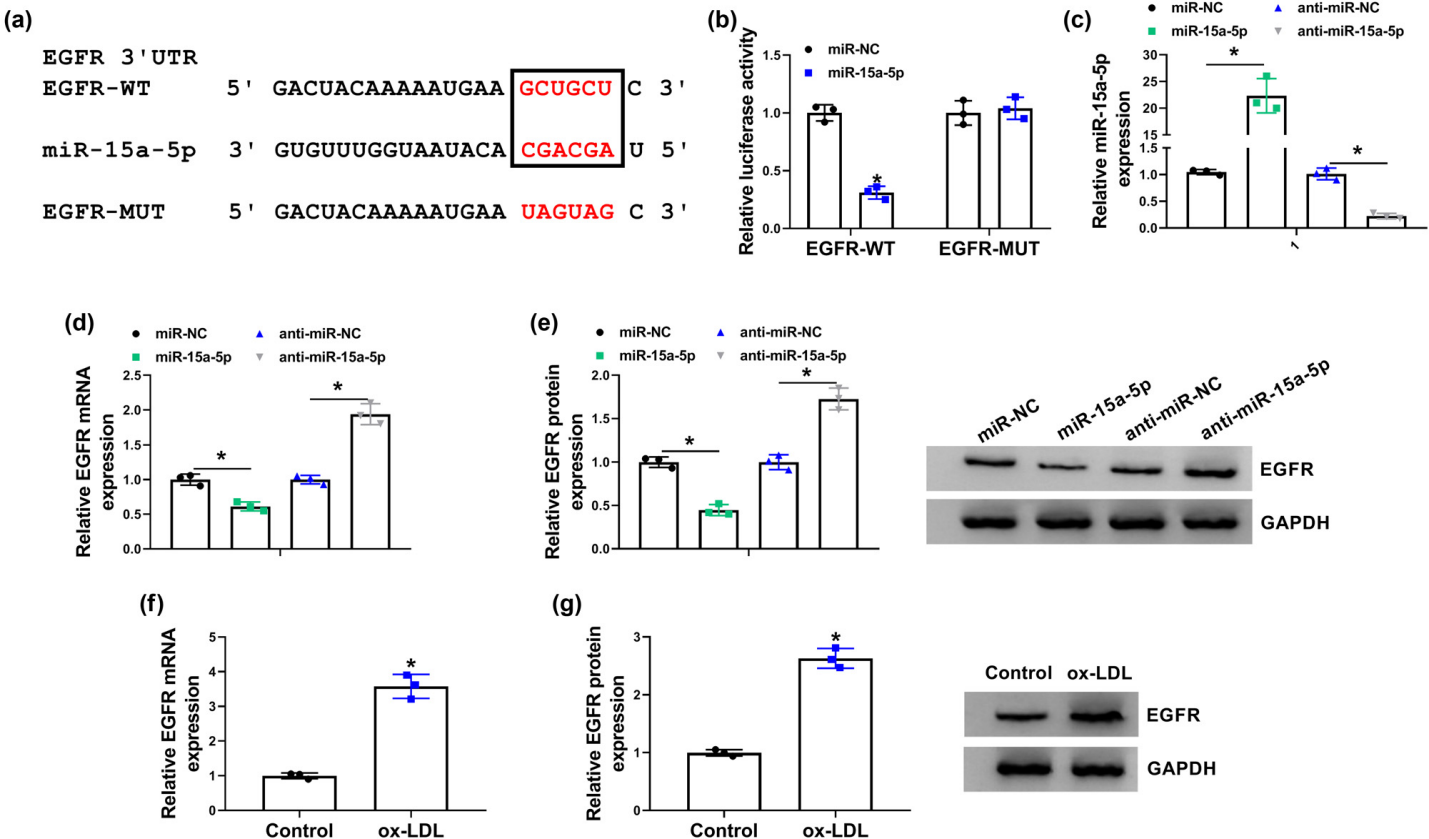
miR-15a-5p interacted with EGFR in HBMECs. (a) The binding sites between miR-15a-5p and EGFR were predicted by starbase3.0 online database. (b) Luciferase activity was detected by dual-luciferase reporter assay. (c) The efficiency of miR-15a-5p mimic and inhibitor in increasing or decreasing miR-15a-5p expression was determined by qRT-PCR. (d and e) The effects of miR-15a-5p mimic and inhibitor on the mRNA and protein levels of EGFR were determined by qRT-PCR and western blot, respectively. (f and g) The impacts of ox-LDL treatment on the mRNA and protein expression of EGFR were severally determined by qRT-PCR and western blot. *P < 0.05.

The target gene of miR-15a-5p was identified in this part. Starbase3.0 online database showed that EGFR 3′UTR contained the binding sites of miR-15a-5p (Figure 5a). Luciferase reporter assay also illustrated that luciferase activity was dramatically inhibited in EGFR-WT and miR-15a-5p group, but there was no dramatic change in EGFR-MUT and miR-15a-5p group (Figure 5b). Subsequently, qRT-PCR results showed that miR-15a-5p expression was dramatically increased by miR-15a-5p mimic and decreased by miR-15a-5p inhibitor (Figure 5c), suggesting that miR-15a-5p mimic and inhibitor were effective in increasing or decreasing miR-15a-5p expression. The mRNA and protein expressions of EGFR were downregulated by miR-15a-5p and were upregulated by miR-15a-5p inhibitor (Figure 5d and e). Furthermore, results showed that the mRNA and protein expressions of EGFR were increased after ox-LDL treatment (Figure 5f and g). These results demonstrated that miR-15a-5p bound to EGFR in HBMECs.

### 3.6 miR-15a-5p mimic contributed to cell proliferation and repressed cell apoptosis and EndoMT by binding to EGFR under ox-LDL treatment in HBMECs

**Figure 6:**
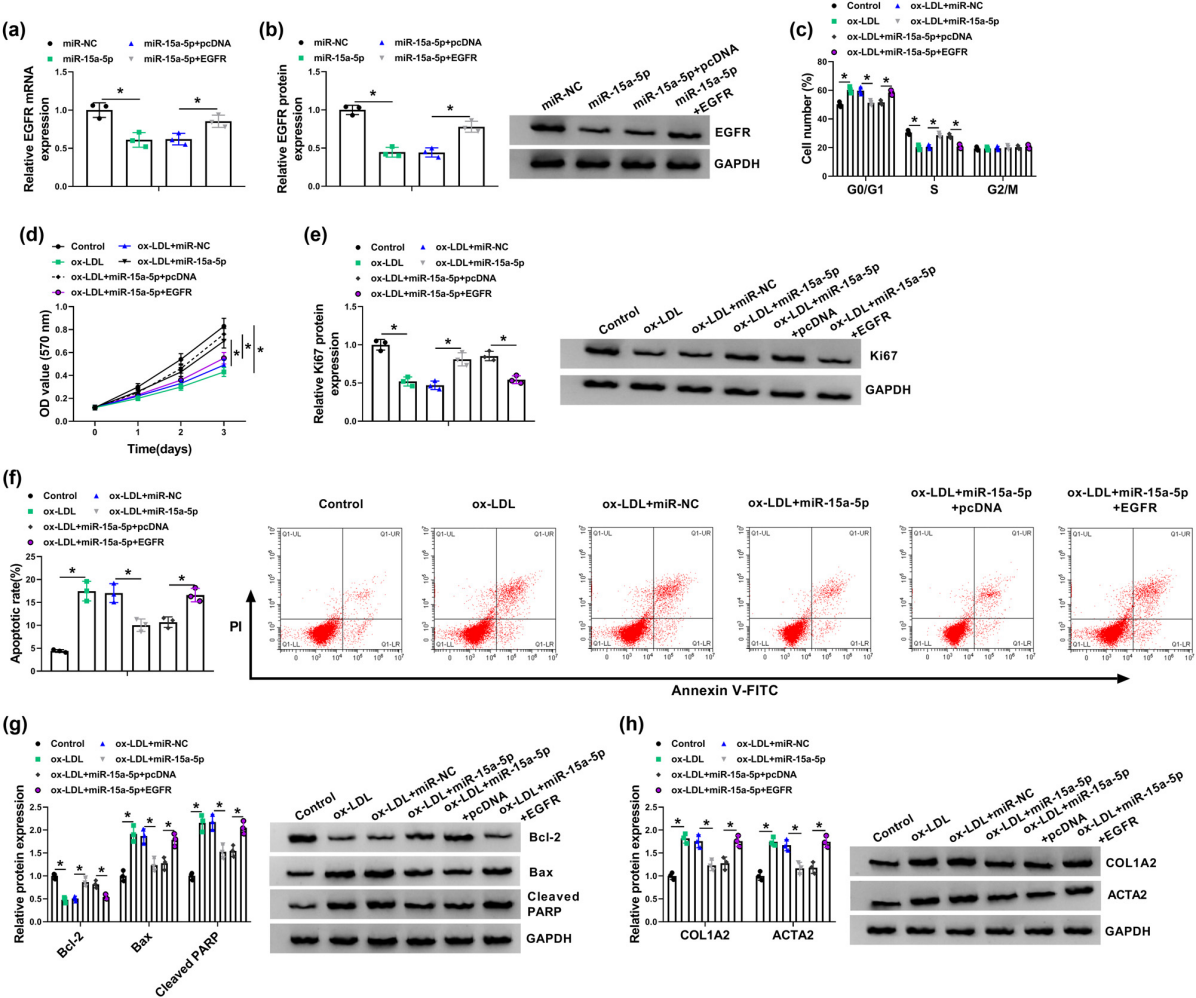
miR-15a-5p mimic contributed to cell proliferation and suppressed cell apoptosis and EndoMT via binding to EGFR in ox-LDL-induced HBMECs. (a and b) The effects between miR-15a-5p and EGFR on the mRNA and protein expression of EGFR were verified by qRT-PCR and western blot, respectively. (c and d) Cell cycle and MTT assays were employed to demonstrate the effects between miR-15a-5p and EGFR on cell proliferation in ox-LDL-induced HBMECs. (e, g, and h) Western blot was employed to disclose the influences between miR-15a-5p and EGFR on the protein expression of Ki67, Bcl-2, Bax, cleaved PARP, COL1A2, and ACTA2 in ox-LDL-induced HBMECs. (f) The impacts between miR-15a-5p mimic and ectopic EGFR expression on the apoptosis of ox-LDL-induced HBMECs were investigated by cell apoptosis assay. *P < 0.05.

Given that miR-15a-5p was associated with EGFR, whether miR-15a-5p regulated ox-LDL-mediated cell proliferation, apoptosis, and EndoMT by interacting with EGFR was further illustrated. First, results showed that the mRNA and protein expression of EGFR was dramatically downregulated by miR-15a-5p, whereas this effect was attenuated by EGFR overexpression (Figure 6a and b). Subsequently, results showed that miR-15a-5p mimic attenuated the promoting effect of ox-LDL treatment on cell cycle arrest, but this impact was restored after EGFR overexpression (Figure 6c). miR-15a-5p mimic also facilitated cell proliferation and Ki67 protein expression under ox-LDL treatment, whereas these effects were attenuated by EGFR overexpression (Figure 6d and e). In addition, miR-15a-5p mimic repressed cell apoptosis after ox-LDL treatment; however, this influence was restored after EGFR transfection (Figure 6f). The protein expression of Bcl-2 was upregulated, and the protein expression of Bax, cleaved PARP, COL1A2, and ACTA2 was downregulated by miR-15a-5p after ox-LDL treatment, but enforced EGFR expression abolished these effects (Figure 6g and h). These results indicated that miR-15a-5p promoted cell proliferation and repressed cell apoptosis and EndoMT by binding to EGFR in ox-LDL-induced HBMECs.

### 3.7 circ_CHFR regulated EGFR expression by sponging miR-15a-5p

**Figure 7:**
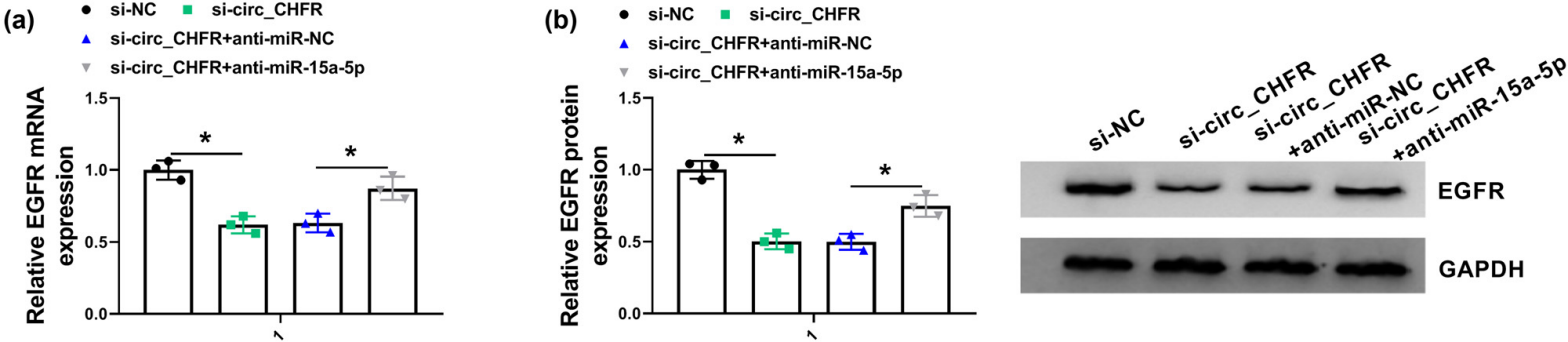
circ_CHFR knockdown downregulated EGFR expression by binding to miR-15a-5p. (a and b) QRT-PCR and western blot were carried out to determine the effects between circ_CHFR silencing and miR-15a-5p inhibitor on the mRNA and protein expression of EGFR, respectively, in HBMEC. *P < 0.05.

This study continued to study whether circ_CHFR regulated EGFR expression by sponging miR-15a-5p. Results showed that the mRNA and protein expression of EGFR were dramatically downregulated by circ_CHFR knockdown, whereas this effect was attenuated after miR-15a-5p depletion in HBMECs (Figure 7a and b). This finding manifested that circ_CHFR regulated EGFR expression via associating with miR-15a-5p in HBMECs.

## 4 Discussion

Brain endothelial cells form the capillary wall and thereby are vital cells in regulating the function of BBB and the balance of brain vessels [26]. Ox-LDL is harmful to many cells, including endothelial cells, and commonly induces endothelial cell injury [27]. Preventing ox-LDL-induced endothelial cell injury becomes a new target in treating vascular diseases [28]. In this study, the effects and underlying mechanism of circ_CHFR in ox-LDL-mediated cell proliferation, apoptosis, and EndoMT in HBMECs are revealed.

Recently, circRNA attracts much attention in regulating endothelial cell development. For example, circ_0003575 knockdown contributed to cell proliferative and angiogenic abilities in human umbilical vein endothelial cells (HUVECs) [15]. Dang et al. reported that circ_0010729 repression inhibited cell proliferative and apoptotic abilities in HUVECs [29]. circ_0029589 (circ_CHFR) was unveiled to suppress cell proliferation and metastasis in vascular smooth muscle [30]. In this study, circ_CHFR was unveiled to regulate cell proliferation, apoptosis, and EndoMT in ox-LDL-induced HBMECs for the first time. First, our finding showed that circ_CHFR expression was dramatically upregulated in ox-LDL-induced HBMECs. Subsequently, to reveal the effects of circ_CHFR on ox-LDL-induced deleterious effects on the HUVEC development, loss-of-function experiments were performed. Results showed that ox-LDL treatment suppressed cell proliferation and accelerated cell arrest at G0/G1 phase, cell apoptosis, and EndoMT, whereas these effects were attenuated after circ_CHFR knockdown. Our results demonstrated that circ_CHFR could regulate ox-LDL-induced deleterious effects on the HUVEC process and circ_CHFR acted as a suppressor in HUVEC development.

The function of miR-15a-5p in ox-LDL-mediated cell proliferation, migration, and EndoMT was also explained in this study. Data have displayed that miR-15a-5p represses cell growth and promotes cell apoptosis in chronic myeloid leukemia [31]; miR-15a-5p overexpression was illustrated to hinder cell proliferation and metastasis in neuroblastoma [32]. In addition, miR-15a-5p was indicated to participate in the progression of colorectal cancer [33], lung cancer [34], and endometrial cancer [35]. In this study, miR-15a-5p was presented to regulate HBMEC development for the first time. In this study, miR-15a-5p was found to bind to circ_CHFR, and its expression was downregulated in ox-LDL-induced HBMECs. In addition, for the sake of revealing the function of miR-15a-5p in HBMEC development, miR-15a-5p inhibitor and si-circ_CHFR were co-transfected into ox-LDL-induced HBMECs. Results showed that miR-15a-5p depletion restored the influences of circ_CHFR silencing on cell proliferation, cell cycle, apoptosis, and EndoMT in ox-LDL-induced HBMECs, suggesting miR-15a-5p contributed to HBMEC development.

miRNA mediates gene expression via associating with mRNA 3′UTR [36]. Thus, the target gene of miR-15a-5p was continued to be predicted. Our result showed that EGFR was a binding gene of miR-15a-5p. Previous researches indicated that EGFR participated in BBB [37] and ischemia [38]. Besides, it was revealed that EGFR was a new target in brain injury [39]. In this study, we found that EGFR expression was significantly increased in ox-LDL-induced HBMECs. Moreover, enforced EGFR expression hindered the impacts of miR-15a-5p on cell proliferation, cell cycle, apoptosis, and EndoMT, meaning that EGFR repressed cell proliferation and contributed to cell apoptosis and EndoMT in ox-LDL-induced HBMECs. Furthermore, to demonstrate whether circ_CHFR regulated EGFR expression via associating with miR-15a-5p, the influences between circ_CHFR depletion and miR-15a-5p inhibitor on EGFR expression were unveiled. Results showed EGFR expression was downregulated by circ_CHFR silencing, which was relieved after miR-15a-5p inhibitor transfection, implicating that circ_CHFR regulated EGFR expression by sponging miR-15a-5p.

Collectively, circ_CHFR and EGFR expression levels were dramatically upregulated, and miR-15a-5p expression was strikingly downregulated in ox-LDL-induced HBMECs. In addition, ox-LDL exposure repressed cell proliferative ability and accelerated cell G0/G1 phase arrest, apoptosis, and EndoMT, whereas circ_CHFR silencing reversed these effects. circ_CHFR was a sponge of miR-15a-5p, and miR-15a-5p inhibitor attenuated the impacts of circ_CHFR downregulation on cell proliferation, cell cycle, apoptosis, and EndoMT in ox-LDL-induced HBMECs. Furthermore, miR-15a-5p bound to EGFR and circ_CHFR regulated EGFR expression via sponging miR-15a-5p. All in all, circ_CHFR silencing restrained the effects of ox-LDL treatment on the proliferation, apoptosis, and EndoMT of HBMECs by miR-15a-5p/EGFR axis. These findings provide a new mechanism for studying the treatment of cerebrovascular diseases.
